# Safe, Efficient, and Comfortable Autonomous Driving Based on Cooperative Vehicle Infrastructure System

**DOI:** 10.3390/ijerph20010893

**Published:** 2023-01-03

**Authors:** Jing Chen, Cong Zhao, Shengchuan Jiang, Xinyuan Zhang, Zhongxin Li, Yuchuan Du

**Affiliations:** 1 Key Laboratory of Road and Traffic Engineering of the Ministry of Education, Tongji University, Shanghai 201804, China; 2 Shanghai Utopilot Technology Co., Ltd., Shanghai 201306, China

**Keywords:** safety, autonomous vehicle, ride comfort, deep reinforcement learning, speed control

## Abstract

Traffic crashes, heavy congestion, and discomfort often occur on rough pavements due to human drivers’ imperfect decision-making for vehicle control. Autonomous vehicles (AVs) will flood onto urban roads to replace human drivers and improve driving performance in the near future. With the development of the cooperative vehicle infrastructure system (CVIS), multi-source road and traffic information can be collected by onboard or roadside sensors and integrated into a cloud. The information is updated and used for decision-making in real-time. This study proposes an intelligent speed control approach for AVs in CVISs using deep reinforcement learning (DRL) to improve safety, efficiency, and ride comfort. First, the irregular and fluctuating road profiles of rough pavements are represented by maximum comfortable speeds on segments via vertical comfort evaluation. A DRL-based speed control model is then designed to learn safe, efficient, and comfortable car-following behavior based on road and traffic information. Specifically, the model is trained and tested in a stochastic environment using data sampled from 1341 car-following events collected in California and 110 rough pavements detected in Shanghai. The experimental results show that the DRL-based speed control model can improve computational efficiency, driving efficiency, longitudinal comfort, and vertical comfort in cars by 93.47%, 26.99%, 58.33%, and 6.05%, respectively, compared to a model predictive control-based adaptive cruise control. The results indicate that the proposed intelligent speed control approach for AVs is effective on rough pavements and has excellent potential for practical application.

## 1. Introduction

Ride comfort has recently received much attention in different driving scenarios due to its influence on the public acceptance of autonomous vehicles (AVs) [1,2] and the health of passengers [3]. Ride comfort is a subjective sensation of passengers associated with the motion of vehicles in different directions. In longitudinal motion, car following is the most frequent scenario. The main task of autonomous car following is maintaining safe and comfortable following gaps via speed control [4]. Regarding vertical motion, the comfort issues caused by dramatic vehicle body vibration on rough pavements are concerned [5]. Speed control helps mitigate vertical vibration on rough pavements. However, safe, efficient, and comfortable speed control is rarely achieved in driving scenarios with car following and rough pavements. Indeed, simultaneously considering pavement conditions and vehicles in front is challenging for a human driver. Heavy congestion and traffic crashes are common on poor roads in peak periods. In this complex driving scenario, intelligent speed control of AVs promises to improve safety, efficiency, and ride comfort and mitigate driver workload.

For car-following behavior, rule-based and supervised learning-based approaches are used to establish car-following models. In rule-based approaches, conventional car-following models are usually used [6]. However, the rule-based approaches involve strong assumptions and simplification, limiting their real-world application. Supervised learning approaches investigate the relationship between dynamic traffic and acceleration selection using extensive expert demonstrations. However, supervised learning only imitates human driving. Although neural networks can generate outputs regardless of inputs, the generalization capability is limited. Thus, for some untrained complex situations, it is difficult for supervised learning-based approaches to find optimal solutions. For speed control on rough pavements, model-based speed planning, such as dynamic programming, is commonly used [5,7]. However, model-based speed planning is also based on strong assumptions of the environment, so it struggles to address changing environments.

The application of model-free DRL algorithms in dynamic traffic scenarios has recently been researched. For example, Zhu et al. trained a DRL-based car-following model using 2000 periods of car following on urban expressways in Shanghai to outperform conventional car-following models [8]. Wu et al. trained a DRL-based differential variable speed limit controller to improve safety, efficiency, and environmental friendliness on freeways [9]. The experimental results show that the controller reduces travel times and CO_2_ emissions. Mao et al. proposed a DRL-based framework to address the taxi dispatch problem with the imbalance of travel demand and taxi supply [10]. The framework outperforms the vanilla policy gradient method and shallow neural networks regarding convergence rate and quality. The above studies suggest that good performance and broad application of model-free DRL algorithms can be achieved in intelligent control.

In DRL-based speed control, a deep deterministic policy gradient (DDPG) algorithm has been widely used. Zhu et al. proposed a DDPG-based speed control for safe, efficient, and comfortable car-following behavior, which outperforms human drivers and model predictive control (MPC) [4]. However, this DDPG model only considered the dynamics of leading and following vehicles. In practice, driving environments are complex. For example, road alignment impacts vehicle dynamics and driving stability, and pavement conditions influence vehicle body vibration. Buechel and Knoll developed a DDPG-based predictive longitudinal controller that directly selects accelerations according to reference speeds and road grades [11]. Subsequently, the authors of this study have used the DDPG algorithm to control the speed with prior knowledge of the dynamic speed limit and comfortable speeds on rough pavements [12]. However, it only provides a solution to a multi-objective speed control problem for an AV without consideration of surrounding vehicles. Since the DDPG-based speed control has the characteristics of fast computation, superior driving performance, and good scalability [4,12], it promises to be a popular speed control approach in the era of autonomous driving. Thus, it is necessary to modify the existing DDPG-based speed control and extend application scenarios.

**Figure 1:**
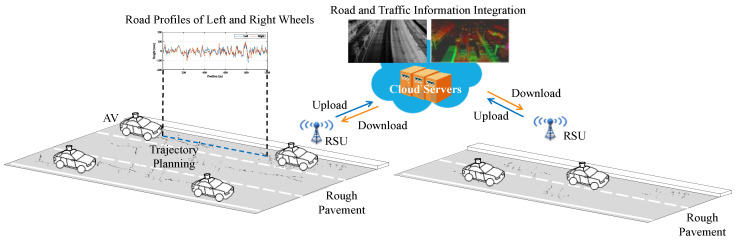
Intelligent speed control for AVs in the CVIS on rough pavements.

In this study, we proposed an intelligent speed control approach for safe, efficient, and comfortable car-following on rough pavements using the DDPG algorithm. As shown in Figure 1, the proposed speed control approach is applied in a cooperative vehicle infrastructure system (CVIS). In this system, AVs detect road profiles using onboard light detection and ranging (LiDAR), accelerometers, and global positioning systems (GPSs) and then send them to roadside units (RSUs) via vehicle-to-infrastructure communication. Dynamic traffic information can be detected by roadside sensors [13,14,15,16,17]. Furthermore, the multi-source road and traffic information is uploaded to the cloud server for integration. When an AV enters the road, it receives complete road profiles of the pavement. The AV then extracts the road profiles of the left and right wheels along the trajectory and calculates comfortable speeds on segments by vertical comfort evaluation. Meanwhile, the AV receives the location and speed information of surrounding vehicles, especially the leading vehicle, via vehicle-to-vehicle communication. Finally, the DRL-based speed control observes the information on comfortable speeds and leading vehicle and recommends accelerations. The AV adjusts driving speed to achieve safe, efficient, and comfortable driving according to recommended accelerations.

The contributions of this study are as follows:

(i) The application of DDPG-based speed control is extended to a scenario with car following and rough pavements, contributing to driving performance improvement and drivers’ workload mitigation in complex driving scenarios.
(ii) A novel reward function is designed by incorporating safety, efficiency, vertical comfort, and longitudinal comfort regarding time to collision, time headway, clearance distance, annoyance rate, jerk, and acceleration.
(iii) The proposed intelligent speed control provides an approach for longitudinal acceleration selection based on dynamic traffic and road information in a CVIS.

The remainder of this paper is organized as follows. Section 2 proposes a vertical comfort evaluation approach using speeds to represent vertical comfort information on oncoming roads. Section 3 presents a DRL-based intelligent speed control for safe, efficient, and comfortable car-following on rough pavements. Section 4 details the training and testing data, DRL model training, and a performance comparison with an MPC baseline. Section 5 summarizes this study’s findings and suggests directions for our future work.

## 2. Vertical Comfort Evaluation

**Figure 2:**
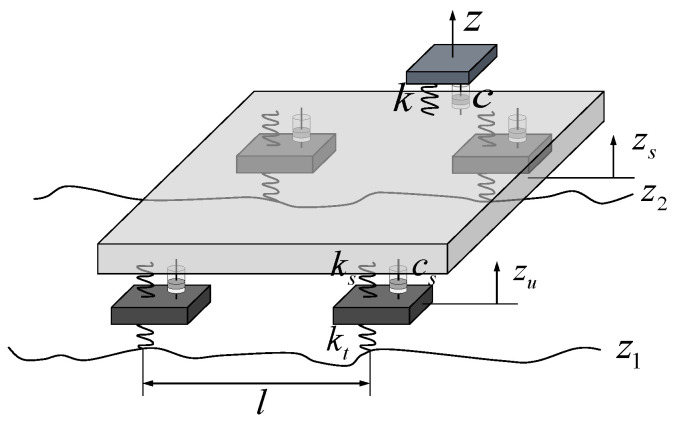
Full-car model.

On rough pavements, irregular road profiles often lead to discomfort in the vertical direction. For a vehicle, vertical ride comfort is directly related to the vertical vibration of the seats, which results from the interactions between the seats, vehicle body, suspensions, tires, wheels, and road profiles. The interactions are formulated as mathematical models [5,7]. Since the most commonly used model, the quarter-car model, is too simple to reflect the entire vibration information, a full-car model with a seat modeling is used (see Figure 2) [7]. The dynamic equation of the full-car model is summarized as

(1) $$[M]\{\ddot{Z}\}+[C]\{\dot{Z}\}+[K]\{Z\}=\{F(t)\},$$

where M, C, and K are the mass matrix, damping matrix, and spring matrix; $\ddot{Z}$, $\dot{Z}$, and Z are the acceleration vector, velocity vector, and displacement vector, respectively. For understanding, Equation (1) is further modified as a state-space formulation:

(2) $$\left\{\begin{array}{l} \ddot{Z}\\ \dot{Z} \end{array}\right\}=\left[\begin{array}{l} -M^{-1}C\;-M^{-1}K\\ \;\;I\;\;\;\;0 \end{array}\right]\left\{\begin{array}{l} \dot{Z}\\ Z \end{array}\right\}+\left[\begin{array}{l} M^{-1}\\ \;\;0 \end{array}\right]F(t)$$

(3) $$F(t)={\left[-k_{t}z_{1}(t),\;-k_{t}z_{2}(t),\;-k_{t}z_{1}\left(t-\frac{l}{v}\right),\;-k_{t}z_{2}\left(t-\frac{l}{v}\right),\;0_{4\times 1}\right]}^{T},$$

where $k_{t}$, $k_{s}$, and k are the stiffness of the tire, suspension, and seat; I is the identity matrix; 0 is the null matrix; $z_{1}$ and $z_{2}$ are the road profiles of the right and left wheels; l is the distance between the front and rear axles; and v is the driving speed. Particularly, the inputs of the full-car model are road profiles in the time domain. Although the spatial road profiles are the same, the time-domain data are changed according to the driving speeds [5]. The values of parameter coefficients are listed in the study of Cantisani and Loprencipe [18].

In the state-space formulation, the output is the acceleration in the time domain with irregular fluctuations. Conversely, the patterns of frequency-domain acceleration are more stable [7]. Hence, the time-domain data are translated into the frequency domain using the power spectral density. In the frequency domain, the vibration in the frequency band 0.5–80 Hz has the largest impact on human sensation, and the effects of the separated bands within this range differ significantly. To distinguish these differences, the evaluation focuses on the vibration in the specific frequency band, and the frequency band is further separated into 23 sections by a 1/3 octave filter [19]. As recommended by ISO 2631-1-1997 [20], the weighted root mean square acceleration (WRMSA) is then used as an objective indicator to evaluate ride comfort. The WRMSA is calculated with a weighting coefficient assigned to each frequency band as

(4) $$a_{w}=\sqrt{\sum_{1}^{23}\omega_{i}^{2}\int_{li}^{ui}S_{\alpha }(f)df},$$

where $\omega_{i}$ is the weighting coefficient for the i-th one-third octave band; ui and li are the upper and lower limiting frequencies of the i-th one-third octave band, respectively; and $S_{\alpha }(f)$ is the power spectral density of the vibration acceleration in the frequency domain.

Although the WRMSA can objectively evaluate ride comfort, the sensitivity differences of passengers cannot be characterized. It is noteworthy that ride comfort is a subjective sensation. Passengers may have distinct feelings even for the same vibration. To represent the proportion of passengers who cannot tolerate the vibration, the annoyance rate in experimental psychology is introduced to modify the evaluation results. The annoyance rate is formulated with random fuzzy evaluation models, membership functions, and probability distributions [19]:

(5) $$A(a_{w})=\int_{x_{\min}}^{\infty }\frac{1}{\sqrt{2\pi }x\sigma }\exp\left[\frac{-{\left[\ln\left(\frac{x}{a_{w}}\right)+0.5\sigma^{2}\right]}^{2}}{2\sigma^{2}}\right]v(x)dx$$

(6) $$\sigma =\sqrt{\ln(1+\delta^{2})}$$

(7) $$v(x)=\left\{\begin{array}{l} 0,\;\;\;\;\;\;\;\;\;\;\;\;\;\;\;x<x_{\min}\\ a\ln(x)+b,\;\;x_{\min}\leq x\leq x_{\max}\\ 1,\;\;\;\;\;\;\;\;\;\;\;\;\;\;\;\;\;x>x_{\max} \end{array}\right.,$$

where $x_{\min}$ is the lower limit of vibration that passengers cannot sense; x is the vibration acceleration; σ is the scale parameter; δ is the vibration parameter ranging from 0.19 to 0.31, generally set as 0.3; a and b are the constants; and $x_{\max}$ is the upper limit of vibration that passengers cannot tolerate. Although the sensation at various magnitudes of vibration depends on passengers’ expectation and activities, ISO 2631-1 proposes an approximate indication of likely reactions to various magnitudes. Based on our previous work [19], $x_{\min}$ and $x_{\max}$ are set as 0.135 and 2.5 m/s^2^, and a and b are 0.4827 and 0.5577.

**Figure 3:**
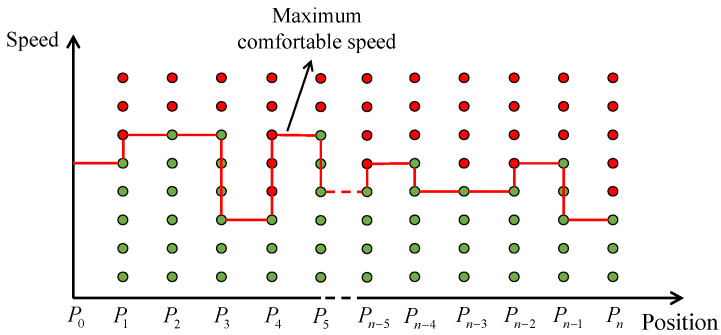
Schematic diagram of MCS calculation.

In this study, the annoyance rate is calculated with a specific length according to conventional road quality evaluation [12]. For example, the road profiles along the driving trajectories are divided into several segments with equal lengths, and the annoyance rate is calculated based on speeds and spatial road profiles in each segment. The intelligent speed control aims to confine the annoyance rate to below 20% to satisfy most passengers [19]. Specifically, the control strategies should ensure that 80% of passengers would be comfortable or not annoyed. The speeds satisfying the standard are regarded as prior knowledge of vertical comfort and directly induce the speed control of AVs. As shown in Figure 3, we calculate the annoyance rates at different speeds and record them at the end of each segment. The green circles indicate annoyance rates below 20%, while the red ones indicate annoyance rates above 20%. The maximum speed on each segment, maintaining the annoyance rate at 20%, is the maximum comfortable speed (MCS). The MCS provides prior knowledge of vertical comfort and works as a reference speed for real-time speed control.

## 3. DRL-Based Intelligent Speed Control

This section proposes a DRL-based intelligent speed control for autonomous car-following on rough pavements. First, we set future road information and current traffic information in the state. We then design a reward function based on speed control objectives. Finally, we present the simulation settings and the structure of the DRL-based speed control model.

### 3.1. State and Action

In DRL, the agent selects an action based on the observed state. The variables in the state should provide sufficient information for the action selection to achieve the control objectives. For safety and efficiency, the relative speed and space between leading and following vehicles should be known. For longitudinal comfort, the previous acceleration limits the current action selection. For vertical comfort, prior knowledge of the MCS along planned driving trajectories provides information on acceptable speeds. Thus, the state is described by the previous acceleration a(t−1), current speed $V_{n}(t)$, relative speed $\Delta V_{n-1,n}(t)$, clearance distance $S_{n-1,n}(t)$, and prior knowledge $V_{p}(t)$ for vertical comfort:

(8) $$s(t)=\left[a(t-1),V_{n}(t),\Delta V_{n-1,n}(t),S_{n-1,n}(t),V_{p}(t)\right],$$

where $\Delta V_{n-1,n}(t)=V_{n}(t)-V_{n-1}(t)$, $V_{n}(t)$ is the speed of the leading vehicle, and $V_{n-1}(t)$ is the speed of the following vehicle (i.e., the AV); the prior knowledge $V_{p}(t)=\left\{V_{p}^{0}(t),V_{p}^{1}(t),\ldots ,V_{p}^{N_{p}}(t)\right\}$ is sampled from the MCS with a certain distance interval to represent future vertical comfort information.

The action is longitudinal acceleration a(t), which is selected in a continuous action space $\left[a_{\min},a_{\max}\right]$; $a_{\min}$ and $a_{\max}$ are the minimum and maximum longitudinal accelerations, set as −3 and 3 m/s^2^, respectively. When the longitudinal acceleration a(t) is given by the agent, the AV’s speed V(t), relative speed $\Delta V_{n-1,n}(t+1)$, and clearance distance $S_{n-1,n}(t)$ are updated in the next timestep:

(9) $$V_{n}(t+1)=V_{n}(t)+a(t)\Delta T$$

(10) $$\Delta V_{n-1,n}(t+1)=V_{n-1}(t+1)-V_{n}(t+1)$$

(11) $$S_{n-1,n}(t+1)=S_{n-1,n}(t)+\frac{\left(\Delta V_{n-1,n}(t)+\Delta V_{n-1,n}(t+1)\right)\Delta T}{2},$$

where ΔT is the simulation sample time interval, usually set as 0.1 s.

### 3.2. Reward Function

In DRL, the agent aims to maximize the expected reward by adjusting the action selection. The reward function plays a crucial role in learning preferred speed control strategies. The reward function should be designed based on the objectives, including safety, efficiency, and ride comfort.

#### 3.2.1. Safety

In dynamic traffic scenarios, safety is the most important element. The time to collision (TTC) is widely used to evaluate the risk of a rear-end crash in real time [21]. The TTC of a following AV is described as

(12) $$TTC(t)=\left\{\begin{array}{l} -\frac{S_{n-1,n}(t)}{V_{n-1,n}(t)},\;V_{n}(t)<V_{n-1}(t)\\ \infty ,\;\;\;\;\;\;V_{n}(t)\geq V_{n-1}(t) \end{array}\right.$$

Specifically, a small TTC value denotes a high traffic crash risk. The TTC threshold should be determined to distinguish unsafe actions. A threshold varying from 1.5 to 5 s is recommended in different studies [4,21]. Based on the experimental results of Zhu et al. [4], the TTC threshold is set as 4 s for a good overall performance. The agent should be punished if the TTC is larger than 0 s and less than 4 s. The TTC feature $R_{st}$ is expressed as

(13) $$R_{st}=\left\{\begin{array}{l} -10,\;\;\;\;\;\;0\leq TTC(t)\leq 4\\ 0,\;\;\;\;\;\;\;\;\;\;\;\;\;\;\;\;otherwise \end{array}\right.$$

Although $R_{st}$ can punish potentially unsafe actions, the TTC values are simultaneously related to clearance distance and relative speed. A lack of sufficient space for emergency braking is also dangerous. Meanwhile, the following AV requires a reaction time for risk assessment, decision-making, and braking. Thus, the safe distance is used as a threshold to ensure sufficient space between vehicles. The agent should be punished when the clearance distance is less than the safe distance. The safe distance feature $R_{sd}$ is described as

(14) $$d_{s}=V_{n-1}(t)\cdot t_{r}+\frac{V_{n}{\left(t\right)}^{2}}{2a_{d}}-\frac{V_{n-1}{\left(t\right)}^{2}}{2a_{d}}$$

(15) $$R_{sd}=\left\{\begin{array}{c} -10,\;\;\;S_{n-1,n}(t)<d_{s}\;\;\;\;\;\\ \;\;\;\;0,\;\;\;S_{n-1,n}(t)\geq d_{s}\; \end{array}\right.,$$

where $t_{r}$ is the reaction time of the following AV, which is set as 1 s in this study; $a_{d}$ is the absolute maximum deceleration.

#### 3.2.2. Efficiency

Efficient driving refers to a short-time headway. Time headway refers to the passed time between leading and following vehicles at a specific point. Maintaining time headway within acceptable limits contributes to a large road capacity. Since the recommended time headway differs between countries, we use the vehicle trajectory data of the Next Generation Simulation (NGSIM) project. A lognormal distribution was fitted based on the extracted car-following events [4]. The reward for driving efficiency uses the probability density function of the lognormal distribution. When the time headway is within the limits, the agent can receive a positive reward, indicating that the time headway is preferred. If the time headway is too large or small, the reward is close to zero. The time headway feature $R_{eh}$ is expressed as

(16) $$R_{eh}=\left.\frac{1}{h\sigma \sqrt{2\pi }}e^{\frac{-(\ln h-\mu )}{2\sigma^{2}}}\right|\mu =0.4226,\sigma =0.4365,$$

where h is the time headway.

Since the training of DRL models usually begins with the random initialization, a large clearance distance should be punished in early training episodes to avoid useless exploration. The agent is thus guided to adjust the speed control policy in time to improve driving efficiency. When the clearance distance is less than the threshold, the time headway is used to evaluate driving efficiency. Otherwise, the agent is punished. The clearance distance feature $R_{ed}$ is described as

(17) $$R_{ed}=\left\{\begin{array}{l} -\frac{S_{n-1,n}(t)}{d_{e}},\;\;\;\;\;\;S_{n-1,n}(t)>d_{e}\\ 0,\;\;\;\;\;\;\;\;\;\;\;\;\;\;\;\;\;\;\;\;S_{n-1,n}(t)\leq d_{e} \end{array}\right.,$$

where $d_{e}$ the threshold of the clearance distance.

#### 3.2.3. Vertical Comfort

As described in Section 2, driving speeds impact vertical comfort, and the MCS provides vertical comfort information on oncoming roads. To confine discomfort, an AV should maintain its speed in the region $\left[0,V_{p}^{0}(t)\right]$, which only causes discomfort to a few passengers. When the driving speed is within this region, the action is acceptable for vertical comfort, and the feature is set as zero. The agent should receive a penalty when the driving speed is outside this region. In the penalty, the speed deviation from $V_{p}^{0}(t)$ is used to guide the driving speed adjustment. The penalty is divided by the desired speed deviation $\Delta V_{e}^{}$, which helps limit the speed deviation below the expected value. The vertical comfort feature $R_{v}$ is constructed as

(18) $$R_{v}=\left\{\begin{array}{l} \;\frac{{\left(V_{p}^{0}(t)-V(t)\right)}^{2}}{\Delta V_{e}{}^{2}},\;\;\;\;\;\;\;V(t)>V_{p}^{0}(t)\\ 0,\;\;\;\;\;\;\;\;\;\;\;\;\;\;\;\;\;\;\;\;\;\;\;\;\;\;\;V(t)\leq V_{p}^{0}(t) \end{array}\right.$$

#### 3.2.4. Longitudinal Comfort

In longitudinal motion, small absolute values of jerk and acceleration contribute to longitudinal comfort [19,22]. Thus, longitudinal comfort is evaluated by the jerk j(t) and acceleration a(t). However, the largest absolute value of acceleration is 3 m/s^2^, while that for jerk is 60 m/s^3^. Since AVs on rough pavements should achieve relatively large acceleration to adapt to changing MCS, we divide jerk and acceleration by different base values for better speed control results. Meanwhile, the jerk is recommended not to exceed 2.94 m/s^3^ to retain longitudinal comfort. Thus, we punish a jerk whose absolute value exceeds 2.94 m/s^3^ with a penalty coefficient φ. The jerk and acceleration features ($R_{lj}$ and $R_{la}$) are described as

(19) $$j(t)=\frac{a(t)-a(t-1)}{\Delta T}$$

(20) $$R_{lj}=-\left\{\begin{array}{l} \;-\varphi \frac{j{\left(t\right)}^{2}}{3600},\;\;\;\;\;\left|\;V(t)\right|\geq 2.94\\ \;-\frac{j{\left(t\right)}^{2}}{3600},\;\;\;\;\;\;\;\left|\;V(t)\right|<2.94 \end{array}\right.$$

(21) $$R_{la}=-\frac{a{\left(t\right)}^{2}}{90}$$

#### 3.2.5. Immediate Reward

For safe, efficient, and comfortable speed control on rough pavements, the immediate reward is the summation of the above reward items with weights:

(22) $$r=w_{1}R_{st}+w_{2}R_{sd}+w_{3}R_{eh}+w_{4}R_{ed}+w_{5}R_{v}+w_{6}R_{lj}+w_{7}R_{la},$$

where $w_{1}$, $w_{2}$, $w_{3}$, $w_{4}$, $w_{5}$, $w_{6}$, and $w_{7}$ are weights. The weights are used to adjust the reward values to a similar magnitude.

### 3.3. DDPG Algorithm

#### 3.3.1. Simulation Settings

Since Lillicrap et al. [23] first proposed the DDPG algorithm, it has been applied in various autonomous driving environments. The driving scenarios mainly include car following [4,24] and lane changing [25]. However, the scenario of driving on real-world rough pavements is seldom considered. Du et al. first used the DDPG algorithm to solve the speed control problem on real-world rough pavements; however, the behavior of the vehicle in front was ignored [12]. Based on the work in [12], we further extend the environment of car-following tasks with rough pavements. Like most DRL algorithms, the DDPG algorithm models the speed control problem using the interactions between agents and environments. In this study, the agent is an AV. The main elements of the environment include rough pavements, leading vehicles, and following vehicles. Rather than raw road profiles detected by sensors, we conduct vehicle vibration simulation and model rough pavements using the MCS corresponding to the road profiles. In such a way, the environment is simplified. We set the leading vehicles’ driving speeds and locations using empirical human data. Since the road profiles and dynamic traffic are usually detected separately, we combine the data from two irrelevant datasets to establish a stochastic environment. The AV’s kinematic model is described in Equations (9)–(11).

To simulate car following on rough pavements, we elaborated on the simulation settings in the environment here. When an AV enters the road, it receives road and traffic information via vehicle-to-infrastructure and vehicle-to-vehicle communication. Since this study focuses on vehicle control strategies, we assume that the AV drives under ideal communication conditions to follow the settings in most studies [12]. Thus, the future MCS and current leading vehicle information are sent to the AV from the environment. Rough pavements and leading vehicles are randomly extracted from the datasets to ensure randomness in the environment. However, the lengths of rough pavements and empirical human data differ considerably. The length of a real-world rough pavement is generally hundreds of meters, while the length of empirical human data is only tens of seconds. Thus, we assume the AV starts at a random location, and the location and speed of the leading vehicle are set according to the sampled car-following event. When the AV reaches the end of the roads or the car-following event ends, the termination condition is satisfied. The initial speed of the AV is set as the speed of the following vehicle for a relatively good beginning to avoid unnecessary exploration [12].

#### 3.3.2. DDPG Structure

**Figure 4:**
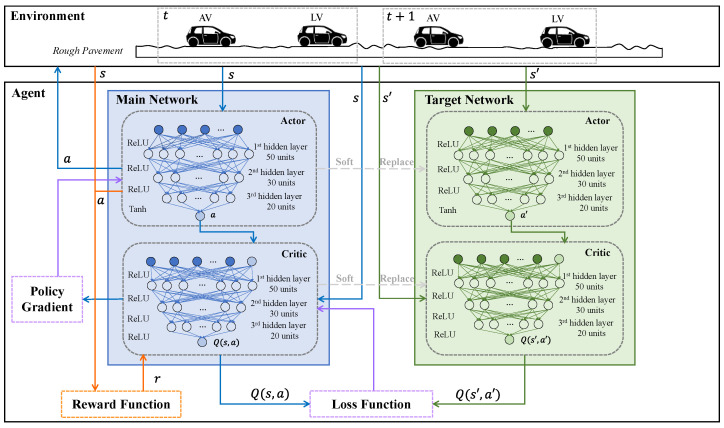
Structure of the DDPG model.

The structure of the DDPG-based speed control model is depicted in Figure 4. The DDPG model comprises two main components: an environment and an agent. The simulation settings illustrated above are used here. The agent has an actor-critic structure. The main and target networks share the same network structure. Specifically, the actor and critic networks in the main network are updated using the policy gradient and loss function in real time, while those in the target networks are updated using soft replacement with the parameters in the main networks. Regarding the structure of networks, the number of layers and neurons is usually selected based on the complexity of the reward function and state. For stable convergence, a large and deep neural network is preferred. A light model is required for a low computational burden and real-world application. Thus, we set the neurons in layers as 50-30-20 units based on extensive trials to balance training performance and computation time. Each neuron in the hidden layer usually uses the ReLU activation function. The final layers in the actor networks use the tanh activation function and are multiplied by 3 to map the output of the actor networks to the range [−3,3].

The actor-network outputs action (longitudinal acceleration) based on the state at each timestep. The action is conducted in the environment and changes the state in the next timestep. The reward is calculated using the reward function proposed in Section 3.2. The transition $\langle s_{t},a_{t},r_{t},s_{t+1}\rangle$ is stored in the experience pool. When the pool is full, network training begins. The training process is described as follows. Initially, the critic and actor networks are initialized. At each timestep t, the actor networks input the state and output an action with a noise: $a_{t}=\mu (s_{t}\left|\theta^{\mu }\right.)+N_{t}$. During training, the noise $N_{t}$ is discounted with a factor. After convergence, the noise should be close to zero.

Although the reward function has punished situations with small TTC values and clearance distances, unsafe actions may still occur. However, unsafe actions are not acceptable in the application. Thus, following the setting in [4], we add a collision avoidance strategy for the action selection in training and testing. When the clearance distance is less than the safe distance, the AV takes a full deceleration of −3 m/s^2^. Otherwise, the action is the output of the actor-network. The collision avoidance strategy is described as

(23) $$a_{t}=\left\{\begin{array}{l} -3,\;\;\;\;\;\;\;\;\;\;\;\;\;\;\;\;\;\;\;\;\;\;\;\;\;\;\;\;\;\;\;\;\;\;\;\;\;\;\;\;\;\;\;\;\;\;\;\;\;\;\;\;\;\;\;\;\;\;\;\;\;\;\;S_{n-1,n}(t)<d_{s}\;\;\;\;\\ \mathrm{DDPG}\;\;\;\mathrm{model}\;\;\mathrm{output},\;\;\;\;\;\;\;\;\;\;\;\;\;\;\;\;\;\;\;\;\;\;\;\;\;\;\;\;otherwise\; \end{array}\right.$$

The critic networks input state $s_{t}$ and action $a_{t}$ and output $Q(s_{t},a_{t})$ to estimate the goodness of the action selection. The main critic network updates by minimizing the loss function L:

(24) $$L=\frac{1}{N}{\sum_{i}\left(r_{i}+\gamma Q^{\prime }\left(s_{i+1},\mu^{\prime }\left(s_{i+1}\left|\theta^{\mu^{\prime }}\right.\right)\left|\theta^{Q^{\prime }}\right.\right)-Q\left(s_{i},a_{i}\left|\theta^{Q}\right.\right)\right)}^{2}$$

where N is the number of samples; r is the reward; γ is the discount factor; $Q\left(s,a\left|\theta^{Q}\right.\right)$ is the main critic network; $\mu \left(s\left|\theta^{\mu }\right.\right)$ is the main actor network; $\theta^{Q}$ and $\theta^{\mu }$ are the parameters of the main critic and actor networks, respectively; $Q^{\prime }\left(s,a\left|\theta^{Q^{\prime }}\right.\right)$ and $\mu^{\prime }\left(s\left|\theta^{\mu^{\prime }}\right.\right)$ are the target critic and actor networks, respectively; $\theta^{Q^{\prime }}$ and $\theta^{\mu^{\prime }}$ are the parameters of the target critic and actor networks, respectively.

The main actor network then updates parameters using the policy gradient $\nabla_{\theta^{\mu }}J$ with the gradients $\nabla_{a}Q(s,a)$ calculated by the main critic network:

(25) $$\nabla_{\theta^{\mu }}J=\frac{1}{N}\sum_{i}\nabla_{a}Q\left(s,a\left|\theta^{Q}\right.\right)\left|{}_{s=s_{i},a=\mu (s_{i})}\right.\nabla_{\theta^{\mu }}\mu \left(s\left|\theta^{\mu }\right.\right)\left|{}_{s_{i}}\right.$$

The target networks are updated slowly by tracking the main networks with τ≪1:

(26) $$\theta^{Q^{\prime }}\leftarrow \tau \theta^{Q}+(1-\tau )\theta^{Q^{\prime }}$$

(27) $$\theta^{\mu^{\prime }}\leftarrow \tau \theta^{\mu }+(1-\tau )\theta^{\mu^{\prime }}$$

## 4. Experiments and Results

In this section, we conduct experiments to show the performance of the proposed intelligent speed control. First, we introduce the dataset for simulating leading vehicles and rough pavements. Then, we train a DDPG model and analyze its training performance. Furthermore, we formulate an MPC-based adaptive cruise control (ACC) as the baseline speed control. The MPC is solved and implemented via CasADi in MATLAB 2020a [26,27]. Finally, we compare the driving performances of the DDPG model and the MPC baseline. All the experiments are executed on a computer with Intel Core i7-5600 at 2.60 GHz and 12 GB RAM.

### 4.1. Data Introduction

To simulate car-following behavior on rough pavements, we use the NGSIM trajectory data and a rough pavement dataset to establish a stochastic environment [4,12]. For an AV, the proposed DRL-based intelligent speed control outputs its acceleration based on the leading vehicle motions, following vehicle (AV) motions, and pavement conditions. During training, the DRL-based intelligent speed control can adjust control strategies adaptively according to changing conditions. In this study, NGSIM trajectory data and the rough pavement dataset are used as an example to train models and verify the feasibility of the proposed intelligent speed control approach. The trajectory and pavement data can be replaced by other datasets.

The NGSIM trajectory data were retrieved from the eastbound I-80 in Emeryville, California, in April 2005. The detection region was 500 m long and covered six lanes. The detection time of the trajectories comprises three spans of time in the afternoon: 4:00–4:15, 5:00–5:15, and 5:15–5:30, which contain the evolutionary process of congestion. The original trajectory data provide locations of vehicles with a detection frequency of 10 Hz. The dataset is reconstructed to enhance the data quality for further investigation, and car-following events are extracted. In this study, 1341 car-following events extracted from the original dataset are used and called the NGSIM data in the following sections. The training set contains 938 events, and the testing dataset contains 403 events.

**Figure 5:**
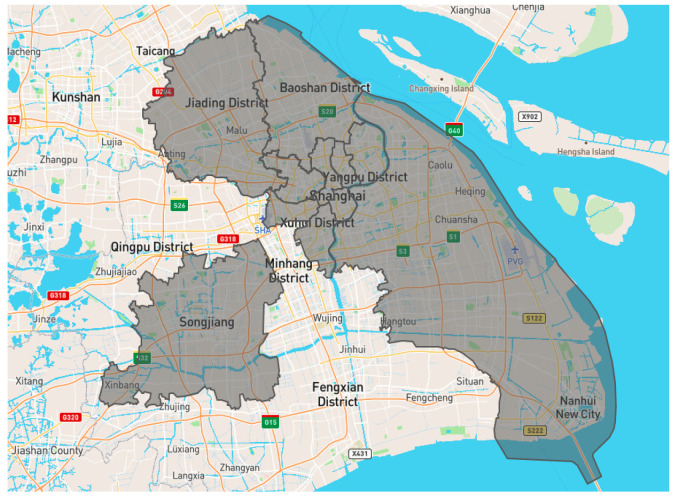
Detection regions of road information.

For pavement data, we collected road information in March and April 2019, covering 11 districts in Shanghai, China (see Figure 5). The road information mainly includes road names, districts, pavement roughness, and road profiles. The road information was detected by advanced onboard sensors, including LiDAR, accelerometers, and GPS, under the operation of manual vehicles. The resolution of detected road profiles detected by LiDAR was 0.25 m. Based on unexpected vibration detected by accelerometers, the potential damage was located by GPS and captured using wavelet analysis [7]. We sampled 110 rough pavements in this dataset to form a rough pavement dataset for model training and testing.

### 4.2. Training Results

We trained a DDPG-based speed control model using the training set of the NGSIM data and rough pavement dataset. At each episode, the environment is reset using data sampled randomly from the datasets, as mentioned in Section 3.3. The preview length of the future MCS is set as 50 m, for example. The resolution of the future MCS is 1 m. According to the definition of the state in Section 3.1, the state has 54 variables. Since training a DRL-based model is time-consuming, the maximum timestep in each episode is set as 1000, and the simulation resolution is 0.1 s. For full exploration, the capacity of the reply buffer is 20,000, and the batch size is 1024. The learning rates of the actor and critic networks are set as 0.0001 and 0.001. The discount factor for calculating the cumulated reward is 0.9. All the weights in Equation (22) are set as 1 to assign equal importance to all the speed control objectives.

**Figure 6:**
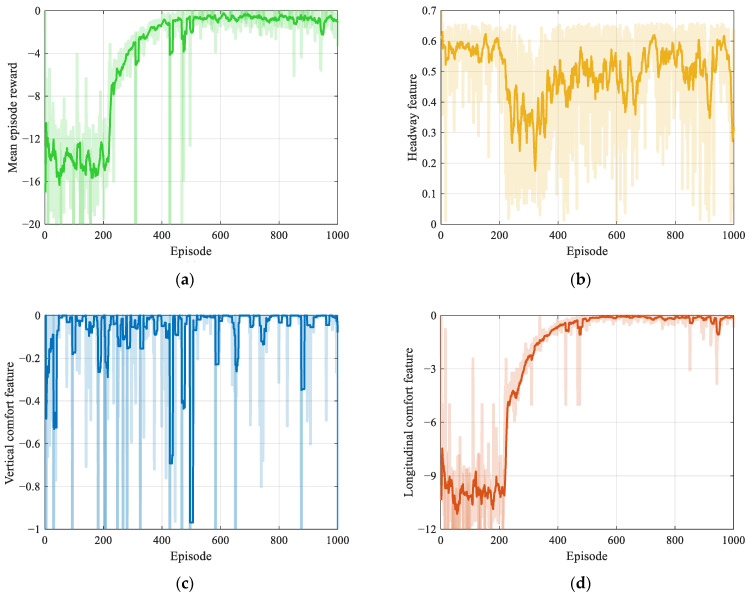
Training trajectories of the DDPG model: (**a**) mean episode reward, (**b**) headway feature, (**c**) vertical comfort feature, and (**d**) longitudinal comfort feature.

Figure 6 illustrates the training process with the episode mean rewards in translucent colors and the rolling mean reward in solid colors. The episode mean reward is the mean value of rewards received in an episode, while the rolling mean reward is the mean value of mean episode rewards within a rolling window. The rolling window is ten episodes. As shown in Figure 6a, the training trajectory of the mean episode reward has a convergence tendency after 400 episodes. In Figure 6b, the headway reward is large in early episodes but decreases later. This is because the agent should balance multiple speed control objectives. Thus, in Figure 6b–d, the longitudinal comfort feature converges after 400 episodes, while there are fluctuations in efficiency and vertical comfort features, indicating that the agent learns longitudinal comfort first and then tries its best to balance comfort and efficiency for higher rewards.

### 4.3. Comparison with MPC

#### 4.3.1. MPC-Based ACC Baseline

MPC is the most common speed control method to achieve multi-objective car-following behavior, including safety, efficiency, and comfort [4,28,29]. At each timestep, MPC solves an optimal control problem in a prediction horizon and generates an acceleration sequence. The first in the sequence is then conducted. This optimization process is repeated until the termination conditions are satisfied. Since MPC-based speed control can handle constraints and perform predictive control, it functions as a baseline for performance comparison with the DDPG model [12]. The kinematic point-mass model mentioned in Section 3.1 is described in a vector form:

(28) x(t+1)=Ax(t)+Bu(t),

where t is the timestep, $x(t)={\left[S_{n-1,n}(t),\Delta V_{n-1,n}(t),V_{n-1,n}(t)\right]}^{T}$, u(t)=a(t), $A=\left[\begin{array}{ccc} 1 & \Delta T & 0\\ 0 & 1 & 0\\ 0 & 0 & 1 \end{array}\right]$, and $B=\left[\begin{array}{c} -0.5\Delta T^{2}\\ -\Delta T\\ \Delta T \end{array}\right]$.

The MPC-based ACC baseline is implemented by optimizing the problem of safe, efficient, and comfortable speed control under constraint conditions. For comparison, the objective function and constraint conditions should refer to the DDPG model. In this study, we follow the modeling of the MPC-based ACC in [4]. For safety and efficiency, AVs follow the leading vehicles with the desired distance ${\tilde{S}}_{n-1,n}$ and a small relative speed $\Delta V_{n-1,n}$. For comfort, the deviations between speed and the current MCS and the absolute jerk and acceleration values should be minimized. Therefore, a constrained MPC formulation is defined as

(29) $$\sum_{t=0}^{N-1}\left[W_{1}{\left(\frac{S_{n-1,n}(t)-{\tilde{S}}_{n-1,n}(t)}{S_{\max}}\right)}^{2}+W_{2}{\left(\frac{\Delta V_{n-1,n}(t)}{\Delta V_{\max}}\right)}^{2}+W_{3}{\left(\frac{V(t)-V_{p}^{0}(t)}{\Delta V_{e}}\right)}^{2}+W_{4}{\left(\frac{j(t)}{j_{\max}}\right)}^{2}\right.\left.+W_{5}{\left(\frac{a(t)}{\alpha }\right)}^{2}\right]$$

(30) s.t. x(t+1)=Ax(t)+Bu(t)

(31) $$\;0<V(t)<V_{\max}$$

(32)  −3<a(t)<3

where N is the prediction horizon (N= 30 in this study); the desired distance ${\tilde{S}}_{n-1,n}(t)=V_{n}(t)h(t)$; and $S_{\max}$, $\Delta V_{\max}$, $\Delta V_{e}$, $j_{\max}$, and α are the constants for normalization. Specifically, $S_{\max}$ and $\Delta V_{\max}$ are the maximum acceptable clearance space and relative speed, set as 15 m and 8 m/s^2^, respectively; $\Delta V_{e}$ is the expected relative speed, set as 3 m/s; $j_{\max}$ is the maximum absolute value of longitudinal jerk, set as 60 m/s^3^; $\alpha^{2}$ is the base value and is set as 90. The weights are set as $W_{1}=1$, $W_{2}=1$, $\;W_{3}=1$, $W_{4}=1$, and $W_{5}=1$; u=[a(0),a(1),…,a(N−1)] is the solved action sequence in each timestep, and only the first action a(0) is implemented. This process is repeated until the termination conditions are reached.

#### 4.3.2. Comparison Results

**Figure 7:**
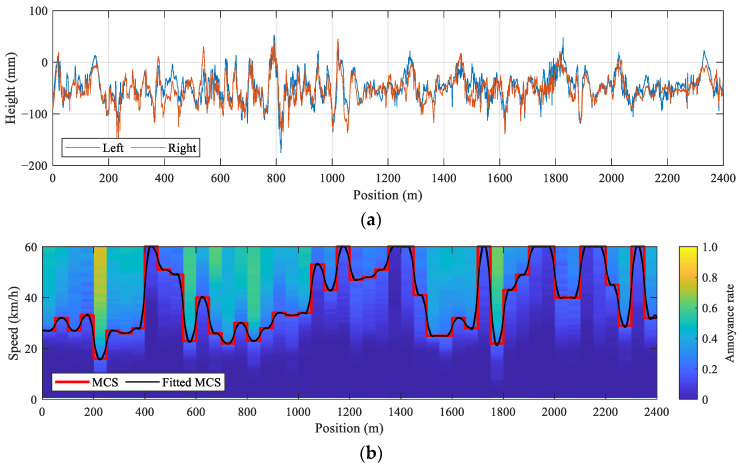
Sampled rough pavement for testing: (**a**) road profiles, and (**b**) annoyance rate and MCS.

To compare driving performances, we conducted experiments using a sampled rough pavement and the testing set of the NGSIM data. Our rationale for this was that 44,330 combinations of rough pavements and leading vehicles exist when AVs start at the same location on each pavement. Since the driving speeds of leading vehicles in the testing set range from 0.0722 to 61.0570 m/s, the deviation between AVs’ speeds and the MCS varies, although the same pavement is used. Since the number of combinations of rough pavements and leading vehicles is large, we sampled an extremely rough pavement from the dataset for testing. The sampled road profiles of left and right wheels, annoyance rate analysis, and MCS of the Yangshupu Road is shown in Figure 7. Specifically, the MCS is fitted using B-spline interpolation to provide precise information for speed tracking, called the fitted MCS [12].

**Figure 8:**
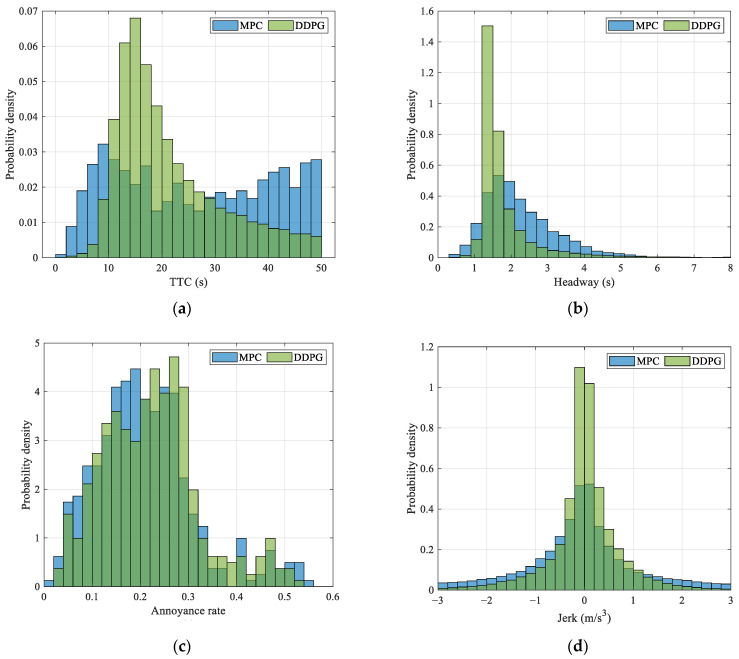
Probability density distribution of (**a**) TTC, (**b**) time headway, (**c**) annoyance rate, and (**d**) jerk.

In the testing, we assume that all the AVs start at a location 0 m on Yangshupu Road, and the leading vehicle is set using the speeds and locations in the testing set. The number of trials is 403. The computation times of the DDPG model and MPC baseline are 125.56 s and 1922.77 s, respectively. Compared to the rolling optimization used in MPC, the DDPG-based speed control exploits linear computations in the networks. The computational efficiency is improved by 93.47%. As shown in Figure 8, we further compare the driving performance using the TTC, time headway, annoyance rate, and jerk. Since the TTC values can be infinity, we pay attention to the TTC values in the region of [0, 50] for analysis and comparison. Similarly, we only show the time headway below 8 s in Figure 8. Figure 8a demonstrates that the MPC baseline has more large TTC values while the DDPG model has a small proportion of small TTC values, indicating that the DDPG model can effectively reduce the risk of rear-end crash and retain safety. Figure 8b shows that the DDPG model has better driving efficiency than the MPC baseline, where almost 80% of the time headway values are less than 2 s. Figure 8c shows that both the DDPG model and MPC baseline can adjust speed according to pavement conditions. Interestingly, the highest annoyance rate of the DDPG model is less than the MPC baseline, but the annoyance rates of the DDPG model on some pavements are slightly larger due to the higher driving efficiency. Figure 8d demonstrates that the DDPG model can limit the absolute value of longitudinal jerk below 2.94 m/s^3^ more effectively, indicating that the DDPG model has better longitudinal comfort. The DDPG model can improve driving efficiency, longitudinal comfort, and vertical comfort by 26.99%, 58.33%, and 6.05%, respectively.

**Figure 9:**
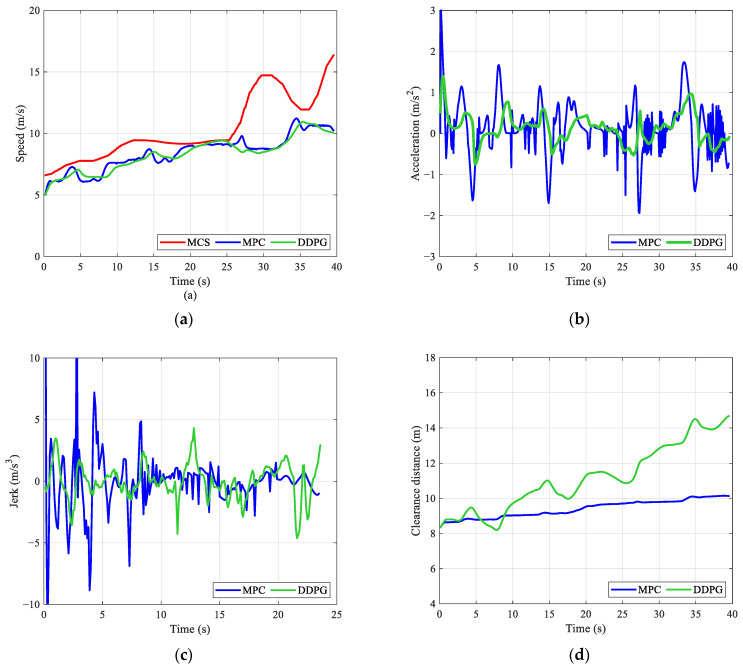
Speed control results for the AV with the leading vehicle No. 107 and a starting point at location 839 m on the sampled rough pavement: (**a**) speed, (**b**) acceleration, (**c**) jerk, and (**d**) clearance distance.

**Figure 10:**
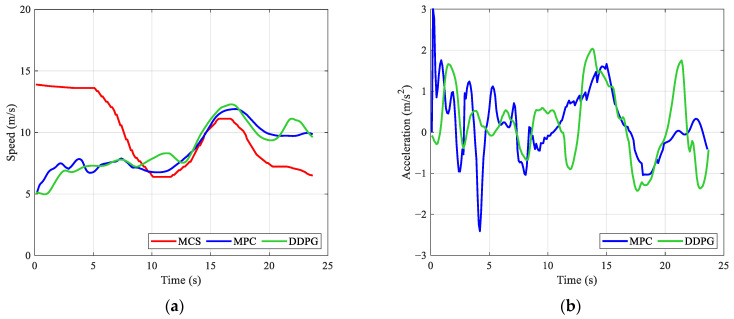

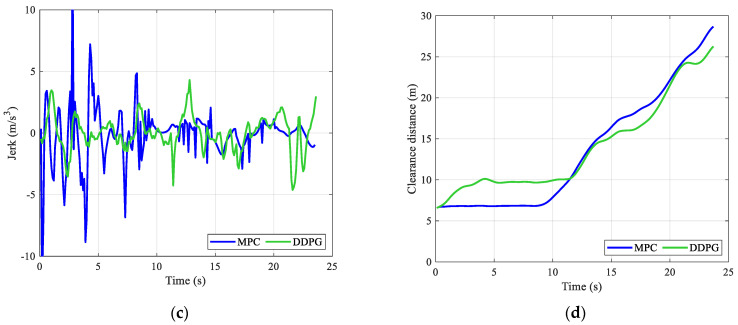
Speed control results for the AV with the leading vehicle No. 326 and a starting point at location 500 m on the sampled rough pavement: (**a**) speed, (**b**) acceleration, (**c**) jerk, and (**d**) clearance distance.

We further tested the model with different starting points to show the details of the speed control results. In Figure 9a, the speeds of the leading vehicle are below the fitted MCS, indicating that the main task of the AV is to follow the leading vehicle. As shown in Figure 9b,c, the DDPG model can generate lower absolute values of jerk and acceleration. Thus, Figure 9a indicates that the speed profile generated by the DDPG model is smoother. Consequently, the space of the DDPG model is much larger than the MPC baseline. Unlike the example in Figure 9, some of the fitted MCS values in Figure 10a are below the leading vehicle’s speeds. The AV should balance driving efficiency and ride comfort. Figure 10d shows that the MPC baseline first follows the leading vehicle at a certain clearance distance and then adjusts its speed to improve vertical comfort. Compared to the MPC baseline, the DDPG model can maintain a relatively large clearance distance for safety. Meanwhile, the DDPG model has lower absolute values of jerk and acceleration when following the leading vehicle. With sufficient space between two vehicles, the AV can adjust its speed in advance for better vertical comfort in future situations (see Figure 10b,c).

## 5. Conclusions

To summarize, this study proposes an intelligent speed control approach for autonomous car following on rough pavements in a cooperative vehicle infrastructure system using deep reinforcement learning (DRL). In experiments, the car-following events in the NGSIM data and road profiles in the rough pavement dataset are used for model training and testing. The experimental results show that the proposed DRL-based speed control has a better driving performance than a model predictive control baseline. Specifically, the DRL-based speed control can improve computational efficiency, driving efficiency, longitudinal comfort, and vertical comfort in car following by 93.47%, 26.99%, 58.33%, and 6.05%, respectively. The results indicate that the proposed intelligent speed control can contribute to autonomous driving on rough pavements and has excellent potential for practical application.

In our future research, we plan to extend driving scenarios with lane-changing behavior. Although lane changing does not have the highest priority in conservative driving strategies, it remains a challenging task with the requirements of safe and comfortable trajectory planning [25,30]. Meanwhile, the proposed intelligent speed control approach can be applied to several AVs with multi-agent RL and used to improve the driving performance in an environment of fully or partially AVs [31]. Moreover, transfer learning and ensemble learning can be used to improve the training efficiency, robustness, and reliability of DRL models [7,32].

## Data Availability

Not applicable.

## References

[1] Bellem H., Thiel B., Schrauf M., Krems J.F. (2018). Comfort in Automated Driving: An Analysis of Preferences for Different Automated Driving Styles and Their Dependence on Personality Traits. Transp. Res. Pt. F-Traffic Psychol. Behav..

[2] Paddeu D., Parkhurst G., Shergold I. (2020). Passenger Comfort and Trust on First-Time Use of a Shared Autonomous Shuttle Vehicle. Transp. Res. Pt. C-Emerg. Technol..

[3] Sharma R.C., Sharma S., Sharma S.K., Sharma N., Singh G. (2021). Analysis of Bio-Dynamic Model of Seated Human Subject and Optimization of the Passenger Ride Comfort for Three-Wheel Vehicle Using Random Search Technique. Proc. Inst. Mech. Eng. Pt. K-J. Multi-Body Dyn..

[4] Zhu M., Wang Y., Pu Z., Hu J., Wang X., Ke R. (2020). Safe, Efficient, and Comfortable Velocity Control Based on Reinforcement Learning for Autonomous Driving. Transp. Res. Pt. C-Emerg. Technol..

[5] Wu J., Zhou H., Liu Z., Gu M. (2020). Ride Comfort Optimization via Speed Planning and Preview Semi-Active Suspension Control for Autonomous Vehicles on Uneven Roads. IEEE Trans. Veh. Technol..

[6] Treiber M., Hennecke A., Helbing D. (2000). Congested Traffic States in Empirical Observations and Microscopic Simulations. Phys. Rev. E.

[7] Du Y., Chen J., Zhao C., Liao F., Zhu M. (2022). A Hierarchical Framework for Improving Ride Comfort of Autonomous Vehicles via Deep Reinforcement Learning with External Knowledge. Comput.-Aided Civ. Infrastruct. Eng..

[8] Zhu M., Wang X., Tarko A., Fang S. (2018). Modeling Car-Following Behavior on Urban Expressways in Shanghai: A Naturalistic Driving Study. Transp. Res. Pt. C-Emerg. Technol..

[9] Wu Y., Tan H., Qin L., Ran B. (2020). Differential Variable Speed Limits Control for Freeway Recurrent Bottlenecks via Deep Actor-Critic Algorithm. Transp. Res. Pt. C-Emerg. Technol..

[10] Mao C., Liu Y., Shen Z.-J.M. (2020). Dispatch of Autonomous Vehicles for Taxi Services: A Deep Reinforcement Learning Approach. Transp. Res. Pt. C-Emerg. Technol..

[11] Buechel M., Knoll A. Deep Reinforcement Learning for Predictive Longitudinal Control of Automated Vehicles. Proceedings of the 2018 21st International Conference on Intelligent Transportation Systems (ITSC).

[12] Du Y., Chen J., Zhao C., Liu C., Liao F., Chan C.-Y. (2022). Comfortable and Energy-Efficient Speed Control of Autonomous Vehicles on Rough Pavements Using Deep Reinforcement Learning. Transp. Res. Pt. C-Emerg. Technol..

[13] Du Y., Qin B., Zhao C., Zhu Y., Cao J., Ji Y. (2022). A Novel Spatio-Temporal Synchronization Method of Roadside Asynchronous MMW Radar-Camera for Sensor Fusion. IEEE Trans. Intell. Transp. Syst..

[14] Zhao C., Song A., Du Y., Yang B. (2022). TrajGAT: A Map-Embedded Graph Attention Network for Real-Time Vehicle Trajectory Imputation of Roadside Perception. Transp. Res. Pt. C-Emerg. Technol..

[15] Zhao C., Song A., Zhu Y., Jiang S., Liao F., Du Y. (2023). Data-Driven Indoor Positioning Correction for Infrastructure-Enabled Autonomous Driving Systems: A Lifelong Framework. IEEE Trans. Intell. Transp. Syst..

[16] Ji Y., Ni L., Zhao C., Lei C., Du Y., Wang W. (2023). TriPField: A 3D Potential Field Model and Its Applications to Local Path Planning of Autonomous Vehicles. IEEE Trans. Intell. Transp. Syst..

[17] Zhao C., Ding D., Du Z., Shi Y., Su G., Yu S. (2023). Analysis of Perception Accuracy of Roadside Millimeter-Wave Radar for Traffic Risk Assessment and Early Warning Systems. Int. J. Environ. Res. Public Health.

[18] Cantisani G., Loprencipe G. (2010). Road Roughness and Whole Body Vibration: Evaluation Tools and Comfort Limits. J. Transp. Eng..

[19] Du Y., Liu C., Li Y. (2018). Velocity Control Strategies to Improve Automated Vehicle Driving Comfort. IEEE Intell. Transp. Syst. Mag..

[20] International Standards Organization (1997). Mechanical Vibration and Shock-Evaluation of Human Exposure to Whole-Body Vibration-Part 1: General Requirements.

[21] Li Y., Xu C., Xing L., Wang W. (2017). Integrated Cooperative Adaptive Cruise and Variable Speed Limit Controls for Reducing Rear-End Collision Risks near Freeway Bottlenecks Based on Micro-Simulations. IEEE Trans. Intell. Transp. Syst..

[22] Hu J., Shao Y., Sun Z., Wang M., Bared J., Huang P. (2016). Integrated Optimal Eco-Driving on Rolling Terrain for Hybrid Electric Vehicle with Vehicle-Infrastructure Communication. Transp. Res. Pt. C-Emerg. Technol..

[23] Lillicrap T.P., Hunt J.J., Pritzel A., Heess N., Erez T., Tassa Y., Silver D., Wierstra D. (2015). Continuous Control with Deep Reinforcement Learning. arXiv.

[24] Yan R., Jiang R., Jia B., Yang D., Huang J. (2021). Hybrid Car-Following Strategy Based on Deep Deterministic Policy Gradient and Cooperative Adaptive Cruise Control. arXiv.

[25] Wang P., Li H., Chan C.-Y. (2019). Continuous Control for Automated Lane Change Behavior Based on Deep Deterministic Policy Gradient Algorithm. Proceedings of the 2019 IEEE Intelligent Vehicles Symposium (IV).

[26] Andersson J.A., Gillis J., Horn G., Rawlings J.B., Diehl M. (2019). CasADi: A Software Framework for Nonlinear Optimization and Optimal Control. Math. Program. Comput..

[27] Zhao C., Liao F., Li X., Du Y. (2021). Macroscopic Modeling and Dynamic Control of On-Street Cruising-for-Parking of Autonomous Vehicles in a Multi-Region Urban Road Network. Transp. Res. Pt. C-Emerg. Technol..

[28] Li S.E., Jia Z., Li K., Cheng B. (2014). Fast Online Computation of a Model Predictive Controller and Its Application to Fuel Economy–Oriented Adaptive Cruise Control. IEEE Trans. Intell. Transp. Syst..

[29] Takahama T., Akasaka D. (2018). Model Predictive Control Approach to Design Practical Adaptive Cruise Control for Traffic Jam. Int. J. Automot. Eng..

[30] Zhao C., Zhu Y., Du Y., Liao F., Chan C.-Y. (2022). A Novel Direct Trajectory Planning Approach Based on Generative Adversarial Networks and Rapidly-Exploring Random Tree. IEEE Trans. Intell. Transp. Syst..

[31] Zhang X., Zhao C., Liao F., Li X., Du Y. (2022). Online Parking Assignment in an Environment of Partially Connected Vehicles: A Multi-Agent Deep Reinforcement Learning Approach. Transp. Res. Pt. C-Emerg. Technol..

[32] Lee K., Laskin M., Srinivas A., Abbeel P. Sunrise: A Simple Unified Framework for Ensemble Learning in Deep Reinforcement Learning. Proceedings of the 38th International Conference on Machine Learning (PMLR).

